# The effect of gradual extinction training on the renewal of electrodermal conditional responses

**DOI:** 10.1111/psyp.14681

**Published:** 2024-09-16

**Authors:** Yi Wang, Camilla C. Luck, Allison M. Waters, Luke J. Ney, Ottmar V. Lipp

**Affiliations:** ^1^ School of Psychology and Counselling, Faculty of Health Queensland University of Technology Brisbane Queensland Australia; ^2^ School of Population Health, Faculty of Health Sciences Curtin University Perth Western Australia Australia; ^3^ School of Applied Psychology, Faculty of Medicine, Dentistry and Health Griffith University Brisbane Queensland Australia

**Keywords:** electrodermal responses, fear conditioning, gradual extinction, renewal, return of fear

## Abstract

Extinction, the repeated presentation of a conditional stimulus (CS) without the unconditional stimulus (US), is the standard paradigm to reduce conditional responding acquired by the repeated pairing of CS and US in acquisition. However, this reduction of conditional responding is prone to relapse. In rodent fear‐conditioning, gradual extinction, the fading out of CS‐US pairings during extinction, has been shown to reduce the return of fear. The current study replicated the gradual extinction procedure in human fear conditioning and assessed whether it reduced the return of fear due to ABA renewal and reacquisition. During extinction, one group received standard extinction, a second received gradual extinction (increasing the spacing of USs presented after the 1st, 3rd, 6th, 10th, and 15th CS+ trials), and a third received reversed extinction training (decreasing the spacing of USs presented after the 1st, 6th, 10th, 13th, and 15th CS+ trials). Larger renewal and faster reacquisition of differential electrodermal responses to CS+ and CS− were expected after standard and reversed extinction than after gradual extinction training. The results were inconclusive due to the failure to find extinction of differential electrodermal responses and US expectancy ratings in both gradual and reversed extinction groups. Despite successful extinction in group standard, renewal was only observed in US expectancy. Visualization of US expectancy ratings during extinction suggested that potential identification of the US presentation patterns during extinction in the gradual and reversed groups delayed extinction learning.

## Abstract 2

Return of fear after successful extinction training is a common observation with broad implications for the success of exposure‐based therapies. In rodents, gradual extinction training has been shown to reduce the return of fear. The current study will translate this approach to human fear conditioning and assess its utility to prevent the return of conditional fear due to changes in context, fear renewal.

## 1 INTRODUCTION

Fear and anxiety disorders pose a major individual and societal burden affecting one in four Australians throughout their lifespan (Australian Institute of Health and Welfare, 2021) and more than 30% of the US population (Vahratian et al., 2021). Given their high prevalence, the availability of effective treatments for these mental health conditions is of utmost importance. At present, cognitive behavioral‐based treatments, frequently involving exposure‐based approaches, are the gold standard treatment (Craske et al., 2022). While effective in reducing fear and anxiety, cognitive behavioral approaches, like most other treatments, have the disadvantage of being associated with high rates of relapse after successful treatment (Loerinc et al., 2015). Thus, the development of interventions that may be less prone to relapse remains a priority.

Basic research aiming to understand the mechanisms involved in exposure‐based treatments has utilized human differential fear conditioning to model outcomes (Lonsdorf et al., 2017; Milad & Quirk, 2012). In human differential fear conditioning, participants are presented with two conditional stimuli (CSs), one (CS+) paired with an aversive unconditional stimulus (US) such as a mild electric shock, and a second (CS−) presented alone. Once the conditioned pairing between CS+ and US has been established, frequently indexed by larger electrodermal responses, fear‐potentiated startles, self‐reported fear, or US expectancy to CS+ relative to CS− (Lipp et al., 2024; Lonsdorf et al., 2017), participants are exposed to extinction training, which involves presentations of the CS+ and CS− in the absence of the US. Return of fear can be assessed using four common approaches: (1) spontaneous recovery, an extinction re‐test presented after a time delay, (2) reinstatement, an extinction re‐test presented after presentations of the US alone, (3) renewal, an extinction re‐test presented in a context different from the extinction context or (4) reacquisition (Bouton, 2002). Extinction training is thought to offer an experimental analogue for exposure‐based interventions (Milad & Quirk, 2012; Vervliet et al., 2013), providing a test for manipulations that can enhance the effectiveness and impact of extinction learning. Theoretically, extinction training is thought to result in a new association between the CS+ and the non‐occurrence of the US that inhibits conditional fear responding and sits alongside the excitatory association between CS+ and US which was acquired during acquisition training (Bouton, 2002).

This conceptualization of extinction learning, as the acquisition of a novel, inhibitory association that leaves the original fear learning unchanged, provides a ready explanation for the high rates of fear relapse observed in clinical practice (Loerinc et al., 2015). After all, the inhibitory association is likely to be weaker than the initial excitatory fear association and is likely to be context‐specific (Bouton, 2002). Past research has provided two potential avenues to enhance the effect of extinction training using (a) manipulations that target the initial fear memory (see for instance memory re‐consolidation, Elsey et al., 2018) and (b) manipulations that strengthen extinction learning. Among the latter approaches (for a review see Lipp et al., 2020), and perhaps counterintuitively, the presentation of additional CS‐US pairings during occasionally reinforced extinction training has been suggested as an effective approach.

### 1.1 Occasionally reinforced extinction training

Bouton et al. (2004) demonstrated the efficacy of occasionally reinforced extinction training in an appetitive conditioning procedure in rodents. After acquisition with eight daily sessions of 24 CS‐US pairings, animals were presented with unreinforced and reinforced extinction trials at a ratio of 6:2 for 8 days and of 1:7 for the next 4 days. Occasionally reinforced extinction led to stronger extinction learning than standard extinction as indicated by slower re‐acquisition. Bouton et al. (2004) proposed a trial signaling theory to explain these findings suggesting that animals learn not only the associations between CS and US but also the relationship between trials. During acquisition, animals learn that CS‐US pairings are always followed by CS‐US pairings, whereas during standard extinction, they learn that CS‐noUS pairings are followed by CS‐noUS pairings. The occasionally reinforced extinction offered the opportunity to learn that CS‐US pairings can be followed by CS‐noUS pairings and CS‐noUS pairings by CS‐US pairings. This learning slowed reacquisition as the presentation of CS‐US pairings did not trigger the expectation of further CS‐US pairings.

Whether occasional presentations of CS‐US pairings presented during extinction training strengthen extinction learning in humans was investigated by Culver et al. (2018). Following acquisition in a continuously reinforced differential conditioning procedure (8 CS ± US, 8 CS−), one group (*n* = 20) underwent standard extinction while a second (*n* = 19) received 6 CS‐US pairings spread evenly among the 24 extinction trials of each CS+ and CS− on day 1 of the experiment. The extinction memory was assessed 1 week after extinction training using spontaneous recovery (4 CS+, 4 CS−), reacquisition (4 CS ± US, 4 CS−), and re‐extinction tests (4 CS+, 4 CS−). The two outcome measures, electrodermal responses and self‐reported US expectancy, were said to provide some evidence for superior extinction learning during partially reinforced extinction training. Electrodermal responses to and US expectancy during CS+ trials increased from the last trial of extinction to the first trial of spontaneous recovery after standard extinction, but not after partially reinforced extinction. However, the latter finding may be due to larger electrodermal responses and increased US expectancy on the last extinction trial after partially reinforced extinction. Moreover, after standard extinction electrodermal responses to the CS+ increased during reacquisition but decreased after partially reinforced extinction. Thompson et al. (2018) employed a single‐day differential conditioning procedure to assess the effects of occasionally reinforced extinction training. Following acquisition (8 CS ± US, 8 CS−), participants (*n* = 24 per group) were presented with standard extinction or 5 CS‐US pairings spread evenly among 24 extinction trials of each CS+ and CS− with the restriction that only one pairing was presented during the last block of 8 CS+ trials (i.e., presented on trial 4). Relative to standard extinction, occasionally reinforced extinction prevented differential electrodermal responding in a subsequent extinction re‐test but did not affect re‐acquisition. Lipp et al. (2021) assessed the effect of occasionally reinforced extinction training on the renewal of differential electrodermal responses in a paradigm that employed a less even spread of 5 CS‐US trials across 19 trials of CS+ alone (2 pairings were presented at random positions within the first 2 blocks of 8 extinction trials and 1 pairing during the last block) after continuously reinforced differential conditioning (8 CS ± US, 8 CS−). Lipp et al. (2021) observed extinction of differential electrodermal responding after standard extinction (*n* = 36), which was followed by reliable renewal, but did not observe extinction after occasionally reinforced extinction (*n* = 34), which may be due to the less predictable distribution of the CS‐US pairings presented in a context that differed from that during acquisition. It should be noted, however, that slowed extinction during occasionally reinforced extinction training was also reported by Culver et al. (2018) and Thompson et al. (2018), although between‐group differences in differential electrodermal responding during the last four trials of extinction were only observed by Culver et al. (2018) but not by Thompson et al. (2018).

### 1.2 Gradual extinction training

Gershman et al. (2013) explicitly addressed the role of the distribution of occasional CS‐US trials during extinction training in their study of gradual extinction in rodents. Using a single cue conditioning procedure, they compared the effects of standard extinction (24 presentations of the CS alone) with gradual extinction (24 presentations of the CS, with increasing spacing of CS‐US pairings on trials 1, 3, 6, 10, and 15) and gradual reverse extinction (24 presentations of the CS, with declining spacing of CS‐US pairings on trials 1, 6, 10, 13 and 15) after continuously reinforced acquisition (3 CS‐US). In comparison to standard extinction and gradual reverse training, spontaneous recovery and reinstatement of conditioned freezing were significantly reduced after gradual extinction training. Gershman et al. (2013) explained their results using a latent state account postulating latent state cues which are encoded with acquisition and extinction memories. Latent state cues represent internal or external events such as the spatiotemporal context that can be used to organize learning into a single or distinct memory episodes. Standard acquisition entails the presentation of USs whereas no USs are presented during extinction. This difference would result in the acquisition and extinction memories being encoded separately which may encourage the return of fear in a novel testing situation. Gradual extinction training removes the sharp separation between acquisition and extinction learning and reduces the encoding of distinct latent cues with acquisition and extinction memories. Gradual extinction enhances the likelihood that the experience of extinction trials would be encoded as part of the acquisition memory. It should be noted that, in contrast to the inhibitory learning account of extinction (Bouton, 2002) this account proposes that extinction training may change the fear memory acquired during acquisition.

Shiban et al. (2015) compared the effects of gradual extinction training (*n* = 16) to standard extinction (*n* = 15) in humans. Following acquisition in a partially reinforced differential conditioning design (16 CS+, 16 CS−; 80% reinforcement schedule), one group of participants was presented with 20 CS+ and CS− trials each in standard extinction whereas the gradual extinction group received CS‐US pairings on CS+ trials 1, 3, 6, 10, and 15. The strength of extinction learning was assessed in a reinstatement test conducted on the next day. The groups did not differ at the end of extinction or in the reinstatement of electrodermal responses or US expectancy, but differential startle potentiation during the reinstatement test was larger after standard extinction. However, this between‐group difference was also present at the end of extinction. Moreover, all participants had a spider picture as the CS+ and a scorpion picture as the CS− without counterbalancing, which may have affected the results (Mallan et al., 2013). van den Akker et al. (2015) assessed the effects of gradual extinction training in an appetitive conditioning procedure (*n* = 30 per group). Following continuously reinforced differential acquisition training (5 CS ± US, 5 CS−), participants were presented during extinction with either 20 trials each of CS+ and CS− alone or had two CS‐US pairings on CS+ trials 2 and 6. Gradual extinction successfully reduced the reacquisition of US expectancy but did not affect the conditioned desire for the US, chocolate mousse, or saliva flow. Finally, Quintero et al. (2021) assessed the effects of gradual extinction in a threat conditioning procedure (Exp 1 and 2: 8 CS ± US, 8 CS−; Exp 3: 7 CS ± US, 1 CS+, 8 CS−) using US expectancy as the sole dependent measure. Across three experiments (*n* = 107, 157, and 75, respectively) Quintero et al. (2021) did not find evidence for differences between standard and gradual extinction (56 CS− trials and 9 CS‐US pairings presented among 47 CS+ trials with no CS‐US pairing during the last 32 trials) on reacquisition or reinstatement tests. In contrast to the animal study conducted by Gershman et al. (2013), the three studies performed in humans yielded quite inconsistent findings. This may be due to changes made to the extinction procedure, for example, Shiban et al. (2015) reduced the total number of extinction trials from 24 to 20 per CS cutting the number of CS+ alone trials after the last CS ± US pairing from 9 to 5, or the overall experimental design. Like most animal studies, Gershman et al. (2013) employed a single cue conditioning procedure whereas the studies within humans employed differential conditioning designs. The added requirement to discriminate between two CSs that is inherent to the differential design as well as the inclusion of a safety signal (CS−) that is never paired with the US may reduce the effectiveness of gradual extinction training. This seems unlikely, however, as studies in humans that assessed the effects of presenting unpaired USs during extinction of differential conditioning yielded the same results as did the single cue studies conducted in rodents (see Bouton et al., 2004; Lipp et al., 2021; Vervliet et al., 2013). Thus, we decided to retain the differential conditioning procedure for the present study.

### 1.3 The present study

Past research in non‐human animals suggests that occasionally reinforced extinction training can enhance extinction learning and reduce the return of fear (Bouton et al., 2004; Gershman et al., 2013), but evidence from human research is mixed. Past work that employed a more even distribution of occasional CS‐US pairings across extinction suggests some evidence for slower reacquisition (Culver et al., 2018) or reduced spontaneous recovery (Thompson et al., 2018), but also for slowed or absent extinction (Lipp et al., 2021) and outcomes varied across dependent measures. Studies using a more gradual distribution of occasional CS‐US pairings during extinction training employed a diverse set of paradigms used and yielded inconsistent results: van den Akker et al. (2015) and Shiban et al. (2015) provided evidence for slowed reacquisition and reduced reinstatement respectively, but Quintero et al. (2021) failed to find evidence for an enhanced effect of gradual extinction training.

To amend this situation and extrapolate from past research, the current study translated Gershman et al.'s (2013) experimental procedure into a human differential fear conditioning design and assessed the effect of gradual extinction training on the renewal and reacquisition of human fear conditioning. All three groups were included: standard extinction, gradual extinction, and reversed gradual extinction. We chose renewal as it seems a more reliable manipulation for the induction of the return of fear (Lipp et al., 2021; Vervliet et al., 2013; Wang et al., 2024) than reinstatement (Sjouwerman & Lonsdorf, 2020) and reacquisition phase was added as past research has yielded evidence for the effectiveness of occasionally reinforced extinction training to slow reacquisition (Bouton et al., 2004;Culver et al., 2018; van den Akker et al., 2015). All other design choices, nature of the CSs and context colors, trial numbers, structure, and sequence of the experimental phases were based on our past research which yielded reliable evidence for renewal of fear conditioning as indexed by electrodermal responses (Lipp et al., 2021, 2024). An ABA renewal paradigm was employed which yields a stronger return of fear than do other designs as it taps the effects of the specificity of acquisition to context A and extinction to context B. Conditioning was assessed using electrodermal responses (Culver et al., 2018; Thompson et al., 2018) and continuous assessments of US expectancies (Culver et al., 2018; van den Akker et al., 2015), indices that provided evidence for between‐group differences in past research. We hypothesized that after gradual extinction renewal would be reduced and reacquisition would be slowed relative to reversed gradual extinction and standard extinction.

## 2 METHOD

### 2.1 Participants

The sample size was determined using G*Power Version 3.1 (Faul et al., 2007) to estimate the power needed to find a CS × Group × Phase interaction analyzing electrodermal first interval responses (FIR) during renewal test based on the effect size of *η* ^2^_p_ = .09 reported by Thompson et al. (2018) for their extinction re‐test. For a mixed design with 3 groups and 4 repeated measures (*F*‐test, ANOVA: repeated measures, within between interaction, *p* = .05, power = .8, sphericity correction = 1, effect size specification = as in SPSS), G*power suggested a total sample size of 51 participants. Each group comprised 36 complete data sets for a full counterbalancing of three factors (i.e., two sets of conditional stimuli, nature of the CS+, and the order of presentation [CS+ first/second]). Participants were recruited from the Queensland University of Technology psychology research participation scheme and the wider university community in exchange for course credit or financial compensation. The sex ratio was controlled to achieve a similar distribution across conditions. One hundred and thirteen first‐year university students were recruited. After excluding four participants due to technical error and one skin conductance non‐responder, a final sample size of 108 participants (87 females) with a mean age of 20.52 (SD = 4.97) was retained for further analyses.

### 2.2 Material and measures

#### 2.2.1 CS and US

The CSs were two sets of pictures of a fish and a bird (Thompson et al., 2018), and each participant was presented with one set. Stimulus set, identity of the CS+, and CS sequence (CS+ or CS− first) were counterbalanced across participants. The US was a sequence of three 2 ms electro‐tactile stimuli generated by a Digitimer DS7A stimulator unit presented 16 ms apart (perceived as one discrete stimulus). US intensity was set to a level that is perceived as “unpleasant, but not painful” in a work‐up procedure for each participant individually. Starting from zero, stimulus intensity was increased in steps of 10 mA until the participants reported noticing the stimulus. These increments were continued until participants report that the stimulus intensity is unpleasant, but not painful. The final stimulation intensity was repeated to confirm the assessment and was used throughout the experiment. The CS+ and CS− were presented for 6 s. The US onset coincided with CS+ offset, but the CS− was never paired with the US. The intertrial intervals, CS offset to CS onset, were set to be 14, 16, or 18 s and varied at random. Stimulus presentation and timing and the collection of self‐report data were controlled by Inquisit 6.6.1 (Inquisit 6 [Computer software], 2022) software.

#### 2.2.2 Physiological measures

Skin conductance was recorded from two pre‐gelled self‐adhesive electrodes (Biopac systems EL507) attached to the thenar and hypothenar eminences of the left hand connected to an EDA100C amplifier as an unfiltered DC signal. To control the effect of deep breaths on skin conductance, respiration was recorded with a Biopac TSD201 transducer attached to the lower torso and connected to an RSP100C respiration amplifier. Electrodermal responses that coincide with extreme deep breaths, sighs, or movement were scored as missing. Physiological measures were recorded at 1000 Hz using a Biopac MP150 unit and using AcqKnowledge Version 3.91 software.

#### 2.2.3 Self‐report measures

Ratings of CS pleasantness, arousal, and fearfulness were recorded using two 9‐point Likert scales ranging from 1 (very unpleasant) to 5 (neutral) to 9 (very pleasant), 1 (very calm) to 5 (neutral) to 9 (very exciting), and 1 (not at all afraid) to 9 (very afraid) respectively, before habituation and after acquisition, extinction, renewal test, and re‐acquisition. The intensity and pleasantness of the US were rated on 11‐point Likert scales with the anchors of 0 (not at all intense) to 10 (very intense) and 0 (very unpleasant) to 10 (very pleasant) after acquisition and extinction. US expectancy was recorded using a continuous rating scale, yielding scores from 10 to 90 (Anchors on display: certain no shock, 2, 3, 4, not sure, 6, 7, 8, certain shock), which was displayed underneath the CS picture on each trial. Participants were asked to select the desired rating using the mouse by moving a pointer from the mid‐point of the scale during the 6 s CS duration. The presentation of the scale coincided with the presentation of the CS.

To control for individual differences that may affect fear conditioning (Lonsdorf & Merz, 2017), participants completed a set of questionnaires after the experiment. The measures included the State–Trait Anxiety Inventory for Adults (STAI; Spielberger et al., 1983) with its two subscales assessing State Anxiety (Cronbach's alpha of .94) and Trait Anxiety (Cronbach's alpha of .93), the Penn State Worry Questionnaire (PSWQ; Meyer et al., 1990; Cronbach's alpha .90), the Mood and Anxiety Symptom Questionnaire (MASQ; Watson et al., 1995) with its two subscales of anxious arousal (Cronbach's alpha of .87) and anhedonic depression (Cronbach's alpha of .94), the Mood and Feelings Questionnaire (MFQ; Angold et al., 1995; Cronbach's alpha .87) and the short version of the Intolerance of Uncertainty Scale (IUS‐12; Carleton et al., 2007; Cronbach's alpha was .89). These measures were included to ensure that the sample is representative for the general population.

### 2.3 Procedure

Ethics approval was granted by the Queensland University of Technology Human Research Ethics Committee (HE Migration Form 2022‐3624‐9316). Upon arrival, participants were guided to the experimental room and provided with information about the experiment. They were informed that pictures of animals would be displayed on a computer screen and that they would receive electro tactile stimuli to their preferred forearm. They were informed where the measurement devices and stimulation electrodes would be attached, that they would be asked to and instructed how to provide expectancy ratings by moving the slide control on the scale presented under the picture to the corresponding labeled number. After any questions were answered and informed consent was provided, the respiration belt, skin conductance electrodes (thenar and hypothenar eminences of the non‐dominant hand), and the stimulation electrode (preferred forearm) were attached. After checking the physiological measurements, the shock workup commenced, followed by the instruction and practice trials on how to use the US expectancy scale. This was followed by a 3 min rest period during which participants were asked to relax and sit quietly without moving too much and an electrodermal baseline was recorded. Before the experimental sequence commenced, the experimenter confirmed the participants' wellbeing and instructed them to “pay attention to the pictures and electro tactile stimuli that will be presented as you will be asked questions about them after the experiment.”

The experimental sequence comprised habituation, acquisition, extinction, renewal test, and reacquisition phases. CS pleasantness, arousal, and fearfulness were rated before habituation. Participants were shown four presentations each of CS+ and CS− in a pseudorandom order in which no more than 2 consecutive trials were of the same stimulus. The first two trials were a CS+ and a CS−, with the stimulus order counterbalanced across participants. There were no electro tactile stimuli presented during habituation. The same counterbalancing rules were applied for all phases of the experiment. The screen background color provided the context stimulus and was manipulated in an ABA sequence with a random allocation of two of three possible colors (blue, yellow, and pink; RGB: 000128255, 255255000, 255128192). One color was used as the background during habituation, acquisition, renewal, and reacquisition (context A), whereas the second (context B) was used during extinction. Context color change (i.e., changing from context A to context B before extinction and context B back to context A after extinction) happened 10 s before the first CS and remained for 5 s after the last CS of each phase. Assessments of self‐reports between phases involved the presentation of the CSs and scales on a black background.

Acquisition followed habituation without interruption and comprised eight presentations each of CS+ and CS−, with the US presented at the offset of the CS+ in a 100% reinforcement schedule. After acquisition, participants provided ratings of CS pleasantness, arousal, fearfulness, and US pleasantness and intensity. Extinction comprised 24 trials each of CS+ and CS−. In the standard extinction group, no US was presented. In the gradual extinction group, CS+ trials 1, 3, 6, 10, and 15 were followed by the US, producing a reduction in US frequency (Gershman et al., 2013). In the reversed group, CS+ trials 1, 6, 10, 13, and 15 were followed by the US, producing an increase in US frequency. After extinction, participants completed CS pleasantness, arousal, fearfulness and US pleasantness, and intensity ratings. Then, renewal was assessed by presenting participants with four trials each of CS+ and CS− followed by ratings of CS pleasantness, arousal, and fearfulness and US pleasantness and intensity ratings. Reacquisition comprised eight presentations each of CS+ and CS−. The CS+ was followed by the US on 50% of the trials (50% reinforcement) with the first CS+ followed by the US and one US randomly allocated to CS+ trials 3–4, 5–6, and 7–8. Participants then provided a final set of CS pleasantness, arousal, and fearfulness and US pleasantness and intensity ratings, had the measurement devices removed and completed the questionnaires. Finally, participants completed the questionnaires and were debriefed, thanked, and awarded course credit or compensation.

### 2.4 Scoring and response definition

The number of spontaneous electrodermal responses displayed during baseline was counted (Dawson et al., 2017). The electrodermal responses elicited by CSs were quantified in three latency windows: (1) the FIR was quantified as the largest response that commenced within 1–4 s of CS onset; (2) the second‐interval response (SIR) was quantified as the largest response that commenced within 4–7 s of CS onset; and (3) the third‐interval response (TIR) was quantified as the largest response that commenced within 7–10 s of CS onset, equivalent to 1–4 s after US onset. The onset of the response starting within each latency window was detected and responses were scored as the difference in skin conductance between response peak and onset without applying a criterion for response duration. Only FIRs were analyzed during habituation (Lipp et al., 2021). TIRs to the US were analyzed only to confirm the equivalence of the groups in electrodermal responding. Electrodermal responses were scored by the experimenter using a custom‐written semi‐automatic interactive program (Ney et al., 2024). The experimenter was blind to the group membership and to the nature of the trial (CS+ or CS−) to be scored. Electrodermal response magnitudes were range corrected to reduce individual differences (Lykken, 1972) and square root transformed to reduce the skew of the distribution (Dawson et al., 2017). US expectancy was measured as the position (10–90) to which the pointer was set by the participant.

### 2.5 Statistical analyses

First‐interval electrodermal responses and US expectancy ratings were the primary dependent variables for the current study. SIRs were a secondary measure that may provide support for the primary electrodermal measure in case of contamination due to enhanced orienting (Luck & Lipp, 2016). Other self‐report measures were accessory and mainly included as manipulation checks. First and second‐interval electrodermal responses and US expectancy ratings from habituation, acquisition, and extinction were subjected to separate 3 (Group: Gradual; Reversed; Standard) × 2 (CS: CS+, CS−) × n (Blocks of two trials) mixed factorial ANOVAs with repeated measures on the last two factors to confirm habituation, acquisition, and extinction of conditional responses. The effect of the experimental manipulation on renewal was assessed in a 3 (Group: Gradual; Reversed; Standard) × 2 (CS: CS+, CS−) × 2 (last trial of extinction, first trial of renewal) mixed factorial ANOVA. The effect of the experimental manipulation on reacquisition was assessed in a 3 (Group: Gradual; Reversed; Standard) × 2 (CS: CS+, CS−) × 4 (Blocks of two trials) mixed factorial ANOVA of the data from re‐acquisition. The results of the multivariate solution (Pillai's trace) were reported as it does not make the assumption of sphericity (Vasey & Thayer, 1987) and the significance level was set to .05. For each analysis, the highest order interaction was followed up with further analyses, *t*‐tests or ANOVAs as required, with adjustment of the overall level of significance.

The CS pleasantness, arousal, and fearfulness ratings were analyzed in 3 (Group: Gradual; Reversed; Standard) × 5 (Phase: Pre‐habituation, Post‐acquisition, Post‐extinction; Post‐renewal; Post‐re‐acquisition) × 2 (CS: CS+, CS−) mixed model factorial ANOVAs, and US ratings were analyzed in 3 (Group: Gradual; Reversed; Standard) × 2 (Phase: Post‐acquisition, Post‐extinction) mixed model factorial ANOVAs. Electrodermal TIRs elicited by the unconditional stimulus during acquisition and extinction were analyzed to provide a manipulation check. Demographic variables, number of spontaneous electrodermal responses during baseline, questionnaire measures, ratings of US intensity and pleasantness and unconditional responses electrodermal responses during acquisition were subjected to one‐way or mixed ANOVAs to confirm that the three groups were equivalent on these measures. One participant was replaced as an electrodermal non‐responder indicating a failure to respond to more than 50% of the unconditional stimuli presented.

## 3 RESULTS

Data sets, SPSS syntax, and descriptive statistics can be found on Open Science Framework at https://osf.io/m2rtk/.

### 3.1 Missing data

Forty datapoints were missing from the total 10,368 datapoints in US expectancy, and 19 were missing from the total 31,104 datapoints in electrodermal responses. The missing electrodermal data were due to reaching the recording threshold and none of the electrodermal responses were excluded for coincidence with extreme deep breaths, sighs, or movement. The missing US expectancy ratings occurred when participants failed to complete ratings before the end of the presentation of the scales. All the missing data were replaced by averages of the corresponding CS+ and CS− trials across all participants.

### 3.2 Manipulation check

One‐way ANOVAs were conducted to compare participants in the three experimental groups and indicated they did not differ in age, *F*(2, 105) = 0.50, *p* = .611, *η* ^2^ = .01, physical intensity of the electro tactile stimulus, *F*(2, 105) = 0.41, *p* = .664, *η* ^2^ = .01, number of spontaneous electrodermal responses during baseline, *F*(2, 105) = 0.58, *p* = .560, *η* ^2^ = .01, or on questionnaire measures (STAI state anxiety, *F*(2, 105) = 0.16, *p* = .853, *η* ^2^ < .01, STAI trait anxiety, *F*(2, 105) = 0.12, *p* = .887, *η* ^2^ < .01, IUS12, *F*(2, 105) = 0.02, *p* = .985, *η* ^2^ < .01, MASQ, *F*(2, 105) = 0.36, *p* = .701, *η* ^2^ = .01, MFQ, *F*(2, 105) = 1.19, *p* = .307, *η* ^2^ = .02, PSWQ, *F*(2, 105) = 0.01, *p* = .989, *η* ^2^ < .01) (Table 1).

**TABLE 1:** Means (standard deviations) of manipulation checks as a function of group.

	Standard	Gradual	Reversed
Age	20.03 (2.89)	20.36 (3.64)	21.17 (7.29)
Shock intensity	95.31 (56.05)	104.31 (77.30)	90.42 (62.64)
Baseline peaks	18.83 (14.87)	22.08 (12.92)	20.94 (10.75)
STAI_SA	43.67 (8.56)	43.58 (10.01)	44.78 (11.30)
STAI_TA	45.33 (10.38)	46.42 (11.29)	46.33 (9.51)
MASQ	91.08 (20.69)	91.06 (22.89)	94.75 (20.41)
PSWQ	56.25 (14.30)	56.36 (15.65)	55.86 (15.54)
MFQ	20.00 (5.37)	21.22 (5.03)	21.92 (5.58)
IUS12	32.50 (9.02)	32.14 (9.32)	32.25 (9.03)

### 3.3 Subjective ratings

**FIGURE 1:**
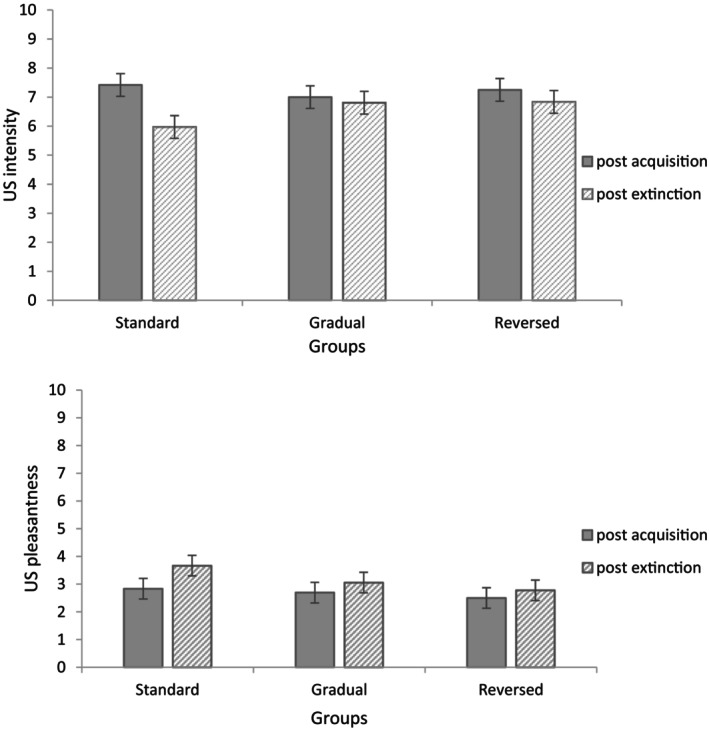
US intensity (tops panel) and US pleasantness (bottom panel) as a function of group and phase (error bars represent the confidence intervals calculated based on the error terms of the within‐subject factor [Masson & Loftus, 2003]).

Two 3 (Group: Gradual; Reversed; Standard) × 2 (Phase: Post‐acquisition, Post‐extinction) mixed factorial ANOVAs were conducted to compare ratings of US intensity and pleasantness respectively (Figure 1). Evaluations of US intensity indicated a main effect of Phase, *F*(1, 105) = 18.03, *p* < .001, *η*_p_^2^ = .15, and a Group × Phase interaction, *F*(2, 105) = 5.69, *p* = .004, *η*_p_^2^ = .10. A significant reduction of intensity rating was found from post‐acquisition to post‐extinction in the standard extinction group, *F*(1, 105) = 26.71, *p* < .001, *η*_p_^2^ = .20, but not in the other groups, both *p*s ≥ .139, with a Bonferroni adjustment (*α* = .017). The group difference might be attributed to a misunderstanding of the question, as participants in the standard extinction group reported selecting “not at all intense” to indicate the absence of the US during extinction. For US pleasantness, there was a main effect of Phase, *F*(1, 105) = 10.32, *p* = .002, *η*_p_^2^ = .09, such that US pleasantness ratings increased from post‐acquisition to post‐extinction.

**FIGURE 2:**
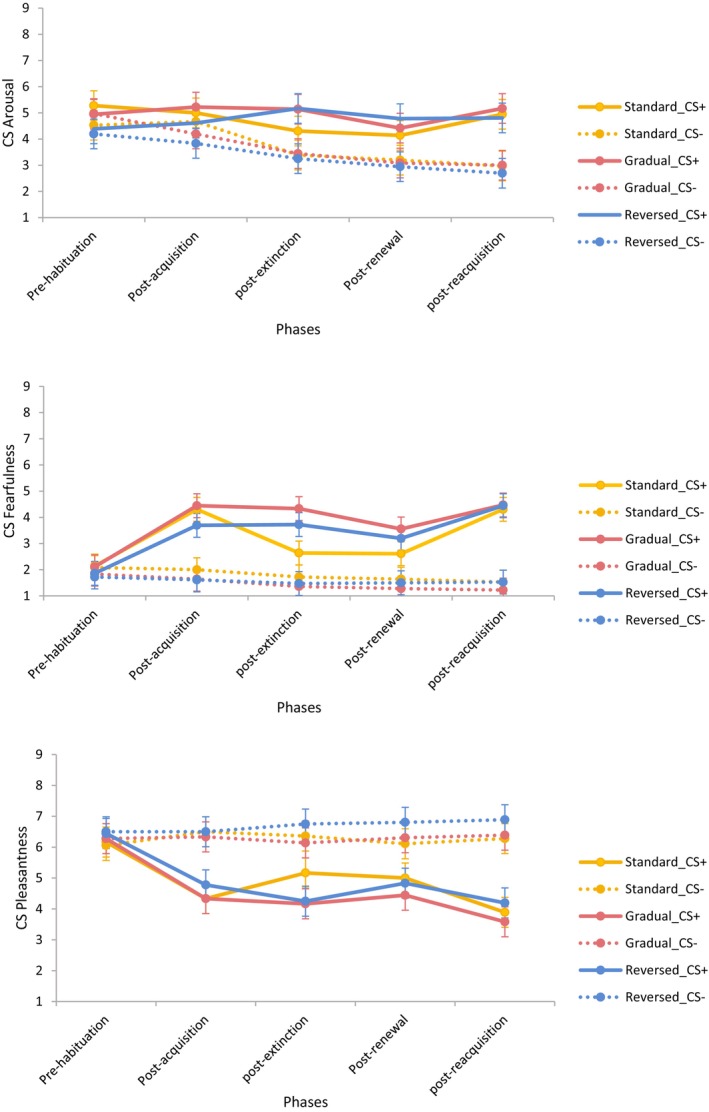
CS arousal (top panel), CS fearfulness (middle panel), and CS pleasantness (bottom panel) as a function of group and phase (error bars represent the confidence intervals calculated based on the error terms of the within‐subject factor [Masson & Loftus, 2003]).

The subjective ratings of CS arousal, fearfulness, and pleasantness were analyzed in 3 (Group: Gradual; Reversed; Standard) × 5 (Phase: Pre‐habituation, Post‐acquisition, Post‐extinction; Post‐renewal; Post‐reacquisition) × 2 (CS: CS+, CS−) mixed model factorial ANOVAs (results shown in Figure 2). All three ANOVAs demonstrated main effects of CS and Phase, and a CS × Phase interaction, all *p*s < .001, with unique Phase × Group (*p* = .003) and Phase × Group × CS interactions (*p* = .008) for CS fearfulness. From post‐acquisition to post‐reacquisition, CS+ was rated as more arousing and less pleasant than CS− averaging across groups, all *p*s ≤ .010 (Bonferroni adjusted *α* = .010). Additionally, a 5 (Phase: Pre‐habituation, Post‐acquisition, Post‐extinction; Post‐renewal; Post‐reacquisition) × 2 (CS: CS+, CS−) ANOVA was conducted for each of the three groups with a Bonferroni adjustment (*α* = .017) to follow up the three‐way interaction found for ratings of fearfulness. In the standard extinction group, there were main effects of CS, *F*(1, 35) = 21.80, *p* < .001, *η*_p_^2^ = .38, Phase, *F*(4, 32) = 5.91, *p* = .001, *η*_p_^2^ = .43, and a Phase × CS interaction, *F*(4, 32) = 6.56, *p* < .001, *η*_p_^2^ = .45. A higher level of fearfulness was reported for CS+ than CS− post‐acquisition and post‐reacquisition, both *p*s *≤* .001 (adjusted *α* = .003). In the gradual extinction group, there were main effects of CS, *F*(1, 35) = 56.41, *p* < .001, *η*_p_^2^ = .62, Phase, *F*(4, 32) = 7.51, *p* < .001, *η*_p_^2^ = .48, and a Phase × CS interaction, *F*(4, 32) = 8.60, *p* < .001, *η*_p_^2^ = .52. A higher level of fearfulness was reported to CS+ than CS− from post‐acquisition to post‐reacquisition, all *p*s *≤* .001 (adjusted *α* = .003). In the reversed extinction group, there were main effects of CS, *F*(1, 35) = 32.87, *p* < .001, *η*_p_^2^ = .48, Phase, *F*(4, 32) = 4.57, *p* = .005, *η*_p_^2^ = .36, and a Phase × CS interaction, *F*(4, 32) = 8.70, *p* < .001, *η*_p_^2^ = .52. A higher level of fearfulness to CS+ than CS− was reported from post‐acquisition to post‐reacquisition, all *p*s *≤* .001 (adjusted *α* = .003).

### 3.4 First interval electrodermal responses

Separate 3 (Group: Gradual; Reversed; Standard) × 2 (CS: CS+, CS−) × n (Blocks of two trials) mixed factorial ANOVAs were conducted for each phase of the experiment. The results for second and TIRs can be found in the Supplementary Materials.

#### 3.4.1 Habituation and acquisition

**FIGURE 3:**
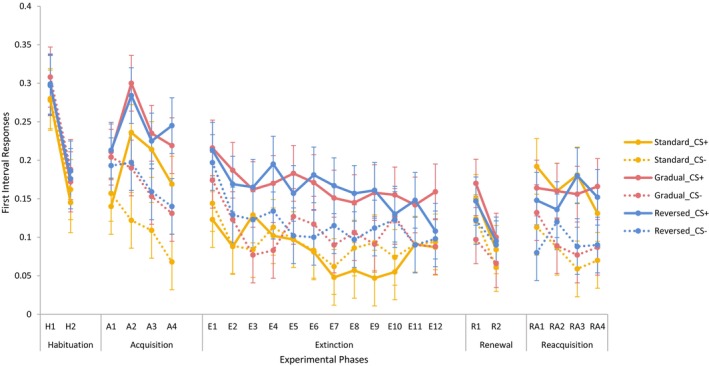
Electrodermal first‐interval responses as a function of group, CS (CS+ vs. CS−), experimental phase, and block (error bars represent the confidence intervals calculated based on the error terms of the within‐subject factor [Masson & Loftus, 2003]).

First interval electrodermal responses across the experimental phases are shown in Figure 3. During habituation, there was a main effect of Block, *F*(1, 105) = 97.68, *p* < .001, *η*_p_^2^ = .48, where the electrodermal responses decreased from the first block to the second. During acquisition, analyses yielded main effects of CS, *F*(1, 105) = 47.71, *p* < .001, *η*_p_^2^ = .31, and Block, *F*(3, 103) = 9.41, *p* < .001, *η*_p_^2^ = .22, and a Block × CS interaction, *F*(3, 103) = 7.81, *p* < .001, *η*_p_^2^ = .19. Averaged across groups, the responses to CS+ was larger than those to CS− in Blocks 2 to 4, all *p*s < .001 (Bonferroni corrected *α* = .013). No effect of group was observed during habituation or acquisition.

#### 3.4.2 Extinction

Analyses found main effects of Block, *F*(11, 95) = 3.60, *p* < .001, *η*_p_^2^ = .29, indicating that the response levels generally declined across blocks (*α* = .004), and CS, *F*(1, 105) = 31.37, *p* < .001, *η*_p_^2^ = .23, and a Group × CS interaction, *F*(2, 105) = 13.47, *p* < .001, *η*_p_^2^ = .20. Averaged across blocks, electrodermal responses were larger to CS+ than to CS− in the gradual extinction, *F*(1, 105) = 36.93, *p* < .001, *η*_p_^2^ = .26, and in the reversed extinction groups, *F*(1, 105) = 20.56, *p* < .001, *η*_p_^2^ = .16, but not in the standard extinction group, *F*(1, 105) = 0.83, *p* = .365, *η*_p_^2^ = .01, with adjusted *α* = .017.

#### 3.4.3 Renewal

Analyses indicated main effects of Block, *F*(1, 105) = 29.38, *p* < .001, *η*_p_^2^ = .22, and CS, *F*(1, 105) = 6.89, *p* = .010, *η*_p_^2^ = .06. Electrodermal responses declined between blocks and were larger to CS+ than to CS−. Effects involving the factor group were absent, all *F*s ≤1.00, *p*s *≥* .371, *η*_p_^2^ ≤ .02.

#### 3.4.4 Reacquisition

There was a main effect of CS, *F*(1, 105) = 42.75, *p* < .001, *η*_p_^2^ = .29, such that CS+ elicited larger responses than CS−. Effects involving the factor group were absent, all *F*s ≤1.35, *p*s *≥* .238, *η*_p_^2^ ≤ .04.

### 3.5 US expectancy

**FIGURE 4:**
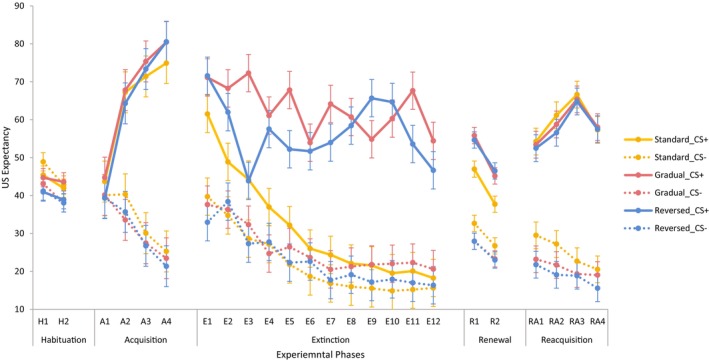
US expectancy as a function of group, CS (CS+ vs. CS−), experimental phase, and block (error bars represent the confidence intervals calculated based on the error terms of the within‐subject factor [Masson & Loftus, 2003]).

The analyses conducted on electrodermal responses were replicated for US expectancy. US expectancy data from the different experimental phases are shown in Figure 4.

#### 3.5.1 Habituation and acquisition

During habituation, there was a main effect of Block, *F*(1, 105) = 8.80, *p* = .004, *η*_p_^2^ = .08, such that Block 1 received higher expectancy ratings than Block 2, and a Group × CS interaction, *F*(2, 105) = 3.17, *p* = .046, *η*_p_^2^ = .06. After alpha adjustment (*α* = .017), differences between CSs failed to reach significance, in groups gradual, *F*(1, 105) = 4.86, *p* = .030, *η*_p_^2^ = .04, standard, *F*(1, 105) = 1.80, *p* = .182, *η*_p_^2^ = .02, and reversed extinction, *F*(1, 105) = 0.03, *p* = .857, *η*_p_^2^ < .01.

During acquisition, analyses yielded main effects of CS, *F*(1, 105) = 224.43, *p* < .001, *η*_p_^2^ = .68, and Block, *F*(3, 103) = 27.60, *p* < .001, *η*_p_^2^ = .45, and a Block × CS interaction, *F*(3, 103) = 80.66, *p* < .001, *η*_p_^2^ = .70. Averaging over groups, US expectancy ratings were higher during CS+ than CS− from block 2 to 4, all *p*s ≤ .001, with a Bonferroni correction (*α* = .013). Effects involving the factor group were absent, all *F*s ≤ 0.94, *p*s *≥* .464, *η*_p_^2^ ≤ .01.

#### 3.5.2 Extinction

Data indicated main effects of CS, *F*(1, 105) = 250.58, *p* < .001, *η*_p_^2^ = .71, Block, *F*(11, 95) = 23.15, *p* < .001, *η*_p_^2^ = .73, and Group, *F*(2, 105) = 27.62, *p* < .001, *η*_p_^2^ = .35, and Group × CS, *F*(2, 105) = 27.22, *p* < .001, *η*_p_^2^ = .34, Block × CS, *F*(11, 95) = 5.11, *p* < .001, *η*_p_^2^ = .37, Group × Block, *F*(22, 192) = 7.73, *p* < .001, *η*_p_^2^ = .47, and Group × Block × CS interactions, *F*(22, 192) = 4.87, *p* < .001, *η*_p_^2^ = .36.

**FIGURE 5:**
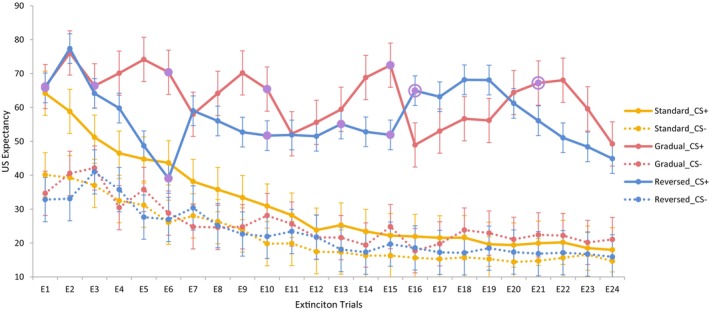
US expectancy ratings during extinction as a function of group, CS (CS+ vs. CS−) and trial (error bars represent the confidence intervals calculated based on the error terms of the within‐subject factor [Masson & Loftus, 2003]). Purple dots indicate the trials with CS‐US pairings, and purple circles highlight the trial with the next CS‐US pairing, if the schedule of presentations were continued.

Following up the three‐way interaction, a 12 (Block) × 2 (CS: CS+, CS−) ANOVA was conducted for each of the three groups, with a Bonferroni correction (*α* = .017). In the standard extinction group, there were main effects of CS, *F*(1, 35) = 16.43, *p* < .001, *η*_p_^2^ = .32, and Block, *F*(11, 25) = 14.83, *p* < .001, *η*_p_^2^ = .87, and a Block × CS interaction, *F*(11, 25) = 3.53, *p =* .004, *η*_p_^2^ = .61. CS+ received higher US expectancy ratings than CS− in Blocks 1, 2, 3, and 5, all *p*s < .001 (adjusted *α* = .001). In the gradual extinction group, there were main effects of CS, *F*(1, 35) = 115.25, *p* < .001, *η*_p_^2^ = .77, such that the US expectancy ratings were higher during CS+ than CS−, and Block, *F*(11, 25) = 5.89, *p* < .001, *η*_p_^2^ = .72, such that the US expectancy ratings showed a general decreasing trend (adjusted *α* = .001), but not the Block × CS interaction, *F*(11, 25) = 2.04, *p* = .068, *η*_p_^2^ = .47. In the reversed extinction group, there were main effects of CS, *F*(1, 35) = 134.58, *p* < .001, *η*_p_^2^ = .79, and Block, *F*(11, 25) = 10.17, *p* < .001, *η*_p_^2^ = .82, and a Block × CS interaction, *F*(11, 25) = 9.44, *p* < .001, *η*_p_^2^ = .81. CS+ received higher US expectancy ratings than CS− in all blocks, all *p*s < .001 (adjusted *α* = .001), with a mean difference of 38.61 in Block 1, dropping to 16.57 in Block 3, climbing up to 48.49 in Block 9, and decreasing to 30.32 in the last block. To further explore the data pattern during extinction, another exploratory analysis was conducted using trials instead of blocks (Figure 5) and the analysis results can be found in the Supplementary Materials.

#### 3.5.3 Renewal

There were main effects of CS, *F*(1, 105) = 129.34, *p* < .001, *η*_p_^2^ = .55, and Block, *F*(1, 105) = 57.14, *p* < .001, *η*_p_^2^ = .35, and Group × CS, *F*(2, 105) = 5.03, *p* = .008, *η*_p_^2^ = .09, and Block × CS interactions, *F*(1, 105) = 11.17, *p* = .001, *η*_p_^2^ = .10. The Group × CS interaction was followed up with Bonferroni corrections (*α* = .017) averaging over blocks. There was significantly higher US expectancy during CS+ than CS− in standard extinction, *F*(1, 105) = 15.82, *p* < .001, *η*_p_^2^ = .13, gradual extinction, *F*(1, 105) = 61.20, *p* < .001, *η*_p_^2^ = .37, and reversed extinction groups, *F*(1, 105) = 62.38, *p* < .001, *η*_p_^2^ = .37, with the CS difference scores of standard (*M*_diff_ = 12.64, SD_diff_ = 18.65), gradual (*M*_diff_ = 24.86, SD_diff_ = 19.20) and reversed groups (*M*_diff_ = 25.10, SD_diff_ = 19.35) failing to reach significance, all *p*s *≥* .020, after Bonferroni correction. The Block × CS interaction was followed up with Bonferroni correction (*α* = .025) averaging across groups. There were significant differences in US expectancy between CS+ and CS− in Block 1, *F*(1, 105) = 123.51, *p* < .001, *η*_p_^2^ = .54, and in Block 2, *F*(1, 105) = 108.57, *p* < .001, *η*_p_^2^ = .51, with a larger difference in Block 1 (*M*_diff_ = 22.96, SD_diff_ = 22.14) than Block 2 (*M*_diff_ = 18.78, SD_diff_ = 19.37), *p* < .001.

#### 3.5.4 Reacquisition

Data showed main effects of CS, *F*(1, 105) = 328.63, *p* < .001, *η*_p_^2^ = .76, and Block, *F*(3, 103) = 10.81, *p* < .001, *η*_p_^2^ = .24, and a Block × CS interaction, *F*(3, 103) = 25.35, *p* < .001, *η*_p_^2^ = .43. Averaging across groups, CS+ was rated significantly higher on US expectancy than CS− in all blocks (adjusted *α* = .013), all *p*s < .001, and differential US expectancy was the smallest in Block 1 (*M*_diff_ = 28.56, SD_diff_ = 22.04), followed by Block 2 (*M*_diff_ = 36.16, SD_diff_ = 22.53) and Block 4 (*M*_diff_ = 39.23, SD_diff_ = 26.08), and largest in Block 3 (*M*_diff_ = 45.30, SD_diff_ = 27.40), all *p*s ≤ .007. Effects involving the factor group were absent, all *F*s ≤1.61, *p*s *≥* .205, *η*_p_^2^ ≤ .03.

### 3.6 Hypothesis testing analyses

The focal hypothesis of differences across groups in renewal was assessed by comparing responses from the last extinction trial and the first renewal trial in a 3 (Group: Gradual, Reversed, Standard) × 2 (CS: CS+, CS−) × 2 (Phase: last extinction trial, first renewal trial) mixed factorial ANOVA.

#### 3.6.1 First interval electrodermal responses

**FIGURE 6:**
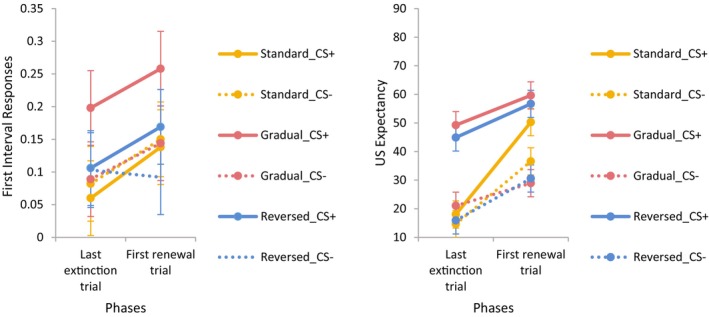
First‐interval electrodermal responses (left panel) and US expectancy (right panel) on the last extinction trial and the first renewal trial as a function of group, CS, and phase (error bars represent the confidence intervals calculated based on the error terms of the within‐subject factor [Masson & Loftus, 2003]).

Electrodermal responses from the last extinction trial and the first renewal trial are shown in the left panel of Figure 6. The analysis yielded a main effect of Phase, *F*(1, 105) = 8.37, *p* = .005, *η*_p_^2^ = .07, such that electrodermal responses increased from the last extinction trial to the first renewal trial, a main effect of CS, *F*(1, 105) = 8.38, *p* = .005, *η*_p_^2^ = .07, and a Group × CS interaction, *F*(2, 105) = 5.78, *p* = .004, *η*_p_^2^ = .10. Averaged across phases, there was a larger electrodermal response to CS+ than to CS− in group gradual extinction, *F*(1, 105) = 17.30, *p* < .001, *η*_p_^2^ = .14, but not in groups standard extinction, *F*(1, 105) = 0.41, *p* = .524, *η*_p_^2^ < .01, or reversed extinction, *F*(1, 105) = 2.23, *p* = .138, *η*_p_^2^ = .02, with *α* = .017. The focal three‐way interaction was not significant, *F*(2, 105) = 0.44, *p* = .645, *η*_p_^2^ = .01.

The renewal effect was not evident in the standard or reversed extinction groups, where there were no differential responses to CS+ and CS− on the last trial of extinction or the first trial of renewal. It appeared that extinction was not evident in the gradual extinction group, as differential responses between CS+ and CS− were observed. Consequently, the effects of gradual and reversed extinction on renewal, as measured by SCRs, remain inconclusive due to unsuccessful extinction and the absence of a baseline for renewal, respectively.

#### 3.6.2 US expectancy

US expectancy data are shown in the right panel of Figure 6. Analyses indicated main effects of CS, *F*(1, 105) = 148.82, *p* < .001, *η*_p_^2^ = .59, Phase, *F*(1, 105) = 106.02, *p* < .001, *η*_p_^2^ = .50, and Group, *F*(2, 105) = 6.75, *p* = .002, *η*_p_^2^ = .11, and Group × CS, *F*(2, 105) = 13.94, *p* < .001, *η*_p_^2^ = .21, and a Group × Phase interactions, *F*(2, 105) = 11.54, *p* < .001, *η*_p_^2^ = .18. The focal three‐way interaction was not significant, *F*(2, 105) = 1.94, *p* = .149, *η*_p_^2^ = .04. The Group × CS interaction was followed up with Bonferroni correction (*α* = .017) averaging over phases. Significant differences were found between CS+ and CS− in standard extinction, *F*(1, 105) = 7.54, *p* = .007, *η*_p_^2^ = .07, gradual extinction, *F*(1, 105) = 90.21, *p* < .001, *η*_p_^2^ = .46, and reversed extinction, *F*(1, 105) = 78.95, *p* < .001, *η*_p_^2^ = .43. The differential responses between CS+ and CS− were larger in the gradual (*M*_diff_ = 29.44, SD_diff_ = 21.78) and reversed (*M*_diff_ = 27.55, SD_diff_ = 18.27) extinction groups compared to the standard extinction group (*M*_diff_ = 8.51, SD_diff_ = 15.16), both *p*s < .001. The Group × Phase interaction was followed up with Bonferroni correction (*α* = .017) averaging across CSs. Significantly higher US expectancy was observed from the last extinction trial to the first renewal trial in standard extinction, *F*(1, 105) = 95.54, *p* < .001, *η*_p_^2^ = .48, gradual extinction, *F*(1, 105) = 10.87, *p* = .001, *η*_p_^2^ = .09, and reversed extinction groups, *F*(1, 105) = 22.69, *p* < .001, *η*_p_^2^ = .18, with a larger increase in group standard extinction (*M*_diff_ = 27.10, SD_diff_ = 18.77) than in groups gradual extinction (*M*_diff_ = 9.14, SD_diff_ = 17.56), *p* < .001, and reversed extinction (*M*_diff_ = 13.20, SD_diff_ = 13.01), *p* = .002.

Although the three‐way interaction was not significant, follow‐up analyses confirmed the presence of renewal in the standard extinction condition with no differential responses between CS+ and CS− at the last trial of extinction and a significant difference at the first renewal trial, *p* < .001 (Figure 4). However, conclusions regarding the effects of gradual and reversed extinction on renewal, as measured by US expectancy, could not be drawn as differential US expectancy was evident in both groups on the last trial of extinction, both *p*s < .001.

## 4 DISCUSSION

Gradual extinction training has been shown to be effective in preventing the return of conditioned fear due to spontaneous recovery and reinstatement in rodents (Gershman et al., 2013) and is suggested to prevent the return of fear due to reinstatement in humans (Shiban et al., 2015). The current study aimed to investigate whether gradual extinction training would prevent the return of fear due to renewal and reacquisition in human participants. Following previous work, we predicted reduced renewal and slower reacquisition after gradual extinction, compared to reversed and standard extinction, indicated by both electrodermal responses and US expectancy ratings. Contrary to our expectations, the results were inconclusive due to the failure to find the extinction of differential electrodermal responses and US expectancy in the gradual extinction group. In the reversed extinction group, differential US expectancy was also present at the end of extinction, and differential electrodermal responses were present when analyzed across extinction blocks but absent on the last extinction trial. The presence of differential electrodermal responses and US expectancy ratings at the last trial of extinction training prevented the assessment of renewal or reacquisition. Extinction of differential electrodermal responses was evident in group standard extinction. Here, evidence for renewal was observed in US expectancy ratings, but not in electrodermal responses, a finding that is not uncommon in human studies of ABA renewal (Wang et al., 2024). Of note, the separation of experimental phases was clearly marked with the subjective ratings presented between phases and the change of the background color. Generalized fear can be observed through the increased response to CS− (i.e., in both FIR and US expectancy) from the end of acquisition to the start of the extinction, and the end of extinction to the start of renewal, which might also contribute to the absence of a renewal effect in electrodermal responses due to the smaller differential responses between CS+ and CS−. Moreover, group differences did not appear during reacquisition, between standard and reversed extinction groups, suggesting no effect of reversed extinction on attenuating reacquisition.

The current study failed to find evidence for extinction in the gradual extinction group. This finding resembles the results reported by Lipp et al. (2021) and Culver et al. (2018). Lipp et al. (2024) presented 5 CS ± US pairings randomly dispersed among 19 CS+ alone extinction trials. Culver et al. (2018) presented 6 CS ± US pairings among 19 CS+ alone extinction trials at regularly spaced intervals. In both studies, differential electrodermal responding was retained across the extinction phase and Culver et al. (2018) did not find a reduction in US expectancy during CS+ in extinction. These findings deviate from those reported by Thompson et al. (2018), who, like Lipp et al. (2021), presented 5 CS ± US pairings among 19 CS+ alone extinction trials. Contrary to Lipp et al. (2021), these pairings were presented equally spaced during extinction with no CS‐US pairing among the last 8 CS+ and CS− extinction trials. This resulted in the absence of differential responding during the last blocks of extinction although differential responding seemed larger than in the standard extinction control group at earlier blocks, however, no statistical analyses were reported to confirm this pattern.

Slow extinction as a result of presenting occasional CS‐US pairings has been reported in paradigms other than fear conditioning. Quintero et al. (2021) assessed gradual extinction in a threat learning paradigm where participants had to report the likelihood that a particular shape was followed by an acoustic stimulus (i.e., US expectancy). Occasional CS ± US pairings were presented at a rate of 5 among the first 8 CS+ extinction trials, 3 among the second 8, 1 among the third 8 and none among the remaining 32 extinction trials. Across three experiments, extinction of US expectancy was slower in the gradual extinction groups, compared to standard extinction. Inspection of the Figures in this study suggests that differential US expectancy was extinguished after about 10 trials in standard extinction but was maintained until around trial 35 in the gradual extinction groups. van den Akker et al. (2015) similarly investigated appetitive learning using chocolate mousse as a positive food US and compared partially reinforced extinction, 2 CS ± US pairings (after extinction trials 2 and 6) presented among 20 CS+ alone and 20 CS− trials, with standard extinction. Extinction of US expectancy and desire to eat ratings was slowed in the partially reinforced group compared to the standard extinction group. It should be noted that in both studies, extinction was ultimately achieved, however, it took more trials to establish than did standard extinction.

So how can we explain the failure to find extinction of differential electrodermal responses and US expectancy ratings in the gradual extinction group – and to some extent the reverse extinction group? Visualizing the US expectancy ratings during extinction as a function of trials (see Figure 5) can provide some illustration. Following the first CS+ trial of extinction which was followed by a US, US expectancy increased in groups gradual and reversed on trial 2 followed by a decrease on trial 3 as the US was absent on trial 2. In group gradual, trial 3, which was a CS ± US trial, was followed by a gradual increase in US expectancy until the next CS ± US trial, trial 6, which was followed by a steep decrease in US expectancy. This decrease, which is also evident after the remaining reinforced trials (trials 10 and 15), seems to indicate that participants learned that the CS+ trial which followed a CS ± US trial is not reinforced. Moreover, these decreases are followed by a gradual increase in US expectancy as participants expect the presentation of further CS ± US trials. This increase continues after the last CS ± US trial until trial 21 when the next CS ± US trial would have been due, had the gradual pattern been continued. Given there is no CS ± US trial on trials 21 or 22 a final decrease in US expectancy commences. The pattern of US expectancy is different in group reverse where following trial 1, US expectancy decreases till trial 6, the next CS ± US trial. After this, it seems to remain at a fairly high level maintained by the occasional CS ± US trial till trial 15. At trial 16, US expectancy is increased as a CS ± US trial would have been presented were the reversed pattern continued. After a few more trials characterized by high US expectancy, a final decrease commences which does not meet the level of US expectancy during CS− trials. Thus, the trial‐by‐trial pattern of US expectancy in the gradual and reversed groups suggests that participants learned the different regularities at which the CS ± US trials were presented. Moreover, unaware of the actual number of CS‐US pairings scheduled, participants expected additional pairings at trials 21 and 16 respectively which maintained their US expectancy (and presumably electrodermal responses) at a high level. A similar pattern can also be found in Culver et al. (2018).

Based on the above analysis (which concededly is speculative), it appears that the failure to see complete extinction of differential electrodermal responses and US expectancy ratings in the gradual and reverse extinction groups is due to our participants' ability to learn the respective schedules at which the CS ± US pairings are presented and to predict when the next CS ± US paring might be presented. A similar ability may be absent in the rodents employed in the study by Gershman et al. (2013). It remains interesting to speculate what would have happened if further extinction trials had been presented. The decrease in US expectancy seen on the final trials of extinction in group gradual appears rather steep in comparison to that seen during the early trials of standard extinction. This may suggest that once the expected further CS ± US trials do not materialize, extinction may be fast and perhaps more pronounced.

### 4.1 Limitations and recommendations

The main limitation of the current study is its failure to find renewal in differential electrodermal conditioning in the standard extinction group. This finding is inconsistent with past research from our lab (Lipp et al., 2021) but has been reported before (Vervliet et al., 2013; see Wang et al., 2024 for a review). Renewal was evident in US expectancy, however, again a finding that is not uncommon (Wang et al., 2024). A second limitation is in the failure to find complete extinction in differential electrodermal responses and US expectancy in the gradual extinction group. As discussed above, such a finding is not uncommon in studies of human (fear) conditioning that present occasional CS ± US parings during extinction. However, there was a decreasing trend in differential responding across the last few trials of extinction, as observed in Figures 1 and 2, which suggests that increasing the number of extinction trials may achieve such an outcome. Alternatively, as participants had learned the nature of the gradually increasing number of unreinforced trials between CS ± US pairings after the 4th pairing (see Figure 5) omitting the last pairing may also permit a better assessment of the effects of gradual extinction.

### 4.2 Summary

The current study was designed to replicate the experimental design of Gershman et al. (2013) with human participants and to examine the impact of gradual extinction on renewal and reacquisition in human participants. Gradual extinction training significantly slowed extinction, and differential responses to CS+ and CS− persisted until the end of extinction, that is, after 24 extinction trials. As a result, the effects of gradual extinction on renewal and reacquisition remain inconclusive in human fear conditioning. Given the common occurrence of slowed extinction in previous studies involving occasionally reinforced extinction or gradual extinction, we recommend a repeated study with an extended number of extinction trials/fewer CS ± US pairings to address this unresolved issue.

## AUTHOR CONTRIBUTIONS

**Yi Wang:** Conceptualization; formal analysis; investigation; methodology; writing – original draft; writing – review and editing. **Camilla C. Luck:** Conceptualization; funding acquisition; writing – original draft; writing – review and editing. **Allison M. Waters:** Conceptualization; funding acquisition; writing – original draft; writing – review and editing. **Luke J. Ney:** Conceptualization; investigation; methodology; writing – original draft; writing – review and editing. **Ottmar V. Lipp:** Conceptualization; formal analysis; funding acquisition; methodology; supervision; writing – original draft; writing – review and editing.

## FUNDING INFORMATION

This work was supported by the National Health and Medical Research Council (GNT1156490).

## CONFLICT OF INTEREST STATEMENT

Authors have no conflict of interest.

## Supporting information


**Data S1:** Supplementary materials.

## Data Availability

Data sets can be found on Open Science Framework at https://osf.io/m2rtk/.
