# Supplementary material for: The effect of gradual extinction training on the renewal of electrodermal conditional responses
Source: Psychophysiology. 2024 Sep 16;61(12):e14681. doi: 10.1111/psyp.14681 (PMC11579229; doi:10.1111/psyp.14681)
Supplement: Supplementary file 1 — Data S1: Supplementary materials. [file PSYP-61-e14681-s001.docx]

Supplementary materials

Second Interval Responses

The data of electrodermal second interval responses were presented in Figure S1.

Acquisition

Analysis yielded a main effect of CS, *F*(1, 105) = 34.15, *p* < .001, η_p_^2^ = .25, and a Block × CS interaction, *F*(3, 103) = 16.92, *p* < .001, η_p_^2^ = .33. After a Bonferroni correction (*α* = .013), the electrodermal response to CS+ was smaller than to CS- in the first block, *p* = .012, but significantly larger from the second to the fourth blocks, all *p*s ≤ .002, averaging over groups. Effects involving the factor group were absent, all *F*s ≤ 2.73, *p*s *≥* .070, η_p_^2^ ≤ .05.

Extinction

There were main effects of CS, *F*(1, 105) = 46.15, *p* < .001, η_p_^2^ = .31, and Group, *F*(2, 105) = 5.30, *p* = .006, η_p_^2^ = .09, and a Group × CS interaction, *F*(2, 105) = 10.30, *p* < .001, η_p_^2^ = .16. With a Bonferroni correction (*α* = .017), responses were larger to CS+ than CS- in groups gradual extinction, *F*(1, 105) = 49.21, *p* < .001, η_p_^2^ = .32, and reversed extinction, *F*(1, 105) = 17.17, *p* < .001, η_p_^2^ = .14, but not in standard extinction, *F*(1, 105) = 0.37, *p* = .545, η_p_^2^ < .01, averaging over blocks.

Renewal

Data indicated main effects of Block, *F*(1, 105) = 11.33, *p* = .001, η_p_^2^ = .10, CS, *F*(1, 105) = 9.58, *p* = .003, η_p_^2^ = .08, and a Group × Block × CS interaction, *F*(2, 105) = 3.96, *p* = .022, η_p_^2^ = .07. To follow up the three-way interaction, a 2 (Block: Block 1, Block 2) × 2 (CS: CS+, CS-) ANOVA was conducted for each of the three groups, with a Bonferroni correction (*α* = .017). A main effect of Block was found in the standard extinction group, *F*(1, 35) = 6.31, *p* = .017, η_p_^2^ = .15, such that the response levels decreased from Block 1 to Block 2 and a main effect of CS in the gradual extinction group, *F*(1, 35) = 7.04, *p* = .012, η_p_^2^ = .17, where the response levels were higher for CS+ than CS-. No significant main effect or interaction was found in the reversed group, all *p*s ≥ .276. However, none of the Block × CS interactions reached significance in any of the groups after alpha adjustment, even though the interaction in the gradual extinction group was below the unadjusted alpha level (*p* = .044).

Reacquisition

Analyses found main effects of CS, *F*(1, 105) = 37.54, *p* < .001, η_p_^2^ = .26, and Block, *F*(3, 103) = 3.02, *p* = .033, η_p_^2^ = .08. CS+ elicited larger electrodermal responses than CS-, and the response levels were higher in Block 1 than Block 4, *p* = .003 (adjusted *α* = .013). Effects involving the factor group were absent, all *F*s ≤ 0.99, *p*s ≥ .418, η_p_^2^ ≤ .03.

**Figure S1**

*Electrodermal Second Interval Responses as a Function of Group, CS (CS+ vs. CS-), Experimental Phase and Block* *(Error Bars Represent the Confidence Intervals Calculated Based on the Error Terms of the Within-subject Factor (Masson & Loftus, 2003))*

**Third Interval Responses**

The data of electrodermal second interval responses were presented in Figure S2.

Acquisition

Data showed a main effect of CS, *F*(1, 105) = 1433.00, *p* < .001, η_p_^2^ = .93, Block, *F*(3, 103) = 46.04, *p* < .001, η_p_^2^ = .57, and a Block × CS interaction, *F*(3, 103) = 11.43, *p* < .001, η_p_^2^ = .25. After a Bonferroni adjustment (*α* = .013), responses were larger after CS+ than CS- across all blocks, all *p*s < .001, averaging across groups, with the differences of responses after CS+ and CS- larger in Block 1 than other blocks, all *p*s < .001.

Extinction

Analysis yielded main effects of CS, *F*(1, 105) = 375.09, *p* < .001, η_p_^2^ = .78, Block, *F*(11, 95) = 26.76, *p* < .001, η_p_^2^ = .76, and Group, *F*(2, 105) = 24.62, *p* < .001, η_p_^2^ = .32, and Group × CS, *F*(2, 105) = 93.80, *p* < .001, η_p_^2^ = .64, Group × Block, *F*(22, 192) = 11.74, *p* < .001, η_p_^2^ = .57, Block × CS, *F*(11, 95) = 35.69, *p* < .001, η_p_^2^ = .81, and Group × Block × CS interactions, *F*(22, 192) = 16.03, *p* < .001, η_p_^2^ = .65. To follow up the three-way interaction, a 12 (Block) × 2 (CS: CS+, CS-) ANOVA was conducted for each of the three groups with a Bonferroni adjustment (*α* = .017). No significant main effects or interactions were found in the standard extinction group, all *p*s ≥ .042. In the gradual extinction group, there were main effects of CS, *F*(1, 35) = 183.60, *p* < .001, η_p_^2^ = .84, Block, *F*(11, 25) = 15.58, *p* < .001, η_p_^2^ = .87, and a Block × CS interaction, *F*(11, 25) = 30.99, *p* < .001, η_p_^2^ = .93. Responses after CS+ were larger than after CS- in Blocks 1, 2, 3, 5 and 8, all *p*s < .001 (adjusted *α* = .001). In the reversed extinction group, there were main effects of CS, *F*(1, 35) = 254.30, *p* < .001, η_p_^2^ = .88, Block, *F*(11, 25) = 26.79, *p* < .001, η_p_^2^ = .92, and a Block × CS interaction, *F*(11, 25) = 31.04, *p* < .001, η_p_^2^ = .93. A higher level of responses after CS+ than CS- was found in Blocks 1, 3, 5, 7, 8 and 11, all *p*s *≤* .001 (adjusted *α* = .001).

Renewal

There were a main effect of Block, *F*(1, 105) = 4.67, *p* = .033, η_p_^2^ = .04, where the response level was higher in Block 1 than Block 2, and a Group × CS interaction, *F*(2, 105) = 4.56, *p* = .013, η_p_^2^ = .08. Responses were larger after CS+ than CS- in the reversed extinction group, *F*(1, 105) = 10.78, *p* = .001, η_p_^2^ = .09, but not in standard, *F*(1, 105) = 0.71, *p* = .400, η_p_^2^ = .01, or gradual extinction groups, *F*(1, 105) = 0.07, *p* = .789, η_p_^2^ < .01, averaging across blocks with *α* = .017.

Reacquisition

Analyses demonstrated main effects of CS, *F*(1, 105) = 1060.86, *p* < .001, η_p_^2^ = .91, such that participants showed larger responses after CS+ than CS-, and Block, *F*(3, 103) = 5.06, *p* = .003, η_p_^2^ = .13, where responses were larger on Block 1 than on other blocks, all *p*s ≤ .007, with a Bonferroni adjustment of α = .013.

**Figure S2**

*Electrodermal Third Interval Responses as a Function of Group, CS (CS+ vs. CS-), Experimental Phase and Block* *(Error Bars Represent the Confidence Intervals Calculated Based on the Error Terms of the Within-subject Factor (Masson & Loftus, 2003))*

Exploratory Findings: US Expectancy during Extinction by Trial

To further explore the data pattern during extinction, an exploratory analysis was conducted using trials (Figure 5). There were main effects of CS, *F*(1, 105) = 250.58, *p* < .001, η_p_^2^ = .71, Trial, *F*(23, 83) = 13.63, *p* < .001, η_p_^2^ = .79, and Group, *F*(2, 105) = 27.62, *p* < .001, η_p_^2^ = .35, and Group × CS, *F*(2, 105) = 27.22, *p* < .001, η_p_^2^ = .34, Trial × CS, *F*(23, 83) = 5.40, *p* < .001, η_p_^2^ = .60, Group × Trial, *F*(46, 168) = 4.95, *p* < .001, η_p_^2^ = .58, and Group × Trial × CS interactions, *F*(46, 168) = 4.63, *p* < .001, η_p_^2^ = .56. The three way interaction was also found to fit linear, *F*(2, 105) = 12.62, *p* < .001, η_p_^2^ = .19, cubic, *F*(2, 105) = 17.91, *p* < .001, η_p_^2^ = .25, order 6, *F*(2, 105) = 5.06, *p* = .008, η_p_^2^ = .09, order 7, *F*(2, 105) = 23.65, *p* < .001, η_p_^2^ = .31, order 8, *F*(2, 105) = 10.95, *p* < .001, η_p_^2^ = .17, order 9, *F*(2, 105) = 4.08, *p* = .020, η_p_^2^ = .07, order 12, *F*(2, 105) = 4.68, *p* = .011, η_p_^2^ = .08, order 13, *F*(2, 105) = 7.64, *p* < .001, η_p_^2^ = .13, order 14, *F*(2, 105) = 3.56, *p* = .032, η_p_^2^ = .06, order 17, *F*(2, 105) = 3.75, *p* = .027, η_p_^2^ = .07, order, 19, *F*(2, 105) = 5.71, *p* = .004, η_p_^2^ = .10, and order 21 polynomial functions, *F*(2, 105) = 3.98, *p* = .022, η_p_^2^ = .07.

Following up the three-way interaction, a 24 (Trial) × 2 (CS: CS+, CS-) ANOVA was conducted for each of the three groups, with a Bonferroni correction (*α* = .017). In the standard extinction group, there were main effects of CS, *F*(1, 35) = 16.43, *p* < .001, η_p_^2^ = .32, and Trial, *F*(23, 13) = 9.20, *p* < .001, η_p_^2^ = .94. US expectancy ratings were higher to CS+ than to CS-, and gradually decided across trials (adjusted *α* = .001). In the gradual extinction group, there were main effects of CS, *F*(1, 35) = 115.25, *p* < .001, η_p_^2^ = .77, and Trial, *F*(23, 13) = 5.29, *p* = .002, η_p_^2^ = .90, and a Trial × CS interaction, *F*(23, 13) = 3.53, *p* = .011, η_p_^2^ = .86. CS+ received higher US expectancy ratings than CS- in all trials, all *p*s < .001 (adjusted *α* = .001), with the mean differences fluctuating across extinction. In the reversed extinction group, there were main effects of CS, *F*(1, 35) = 134.58, *p* < .001, η_p_^2^ = .79, and Trial, *F*(23, 13) = 5.28, *p* = .002, η_p_^2^ = .90, and a Trial × CS interaction, *F*(23, 13) = 3.73, *p* = .009, η_p_^2^ = .87. CS+ received higher US expectancy ratings than CS- in all trials, all *p*s < .001 (adjusted *α* = .001), with the mean differences fluctuating across extinction.

Exploratory Findings: Excluding Non-learners

To further clarify the inconclusive results of extinction and renewal, a filter to exclude non-learners was applied. The criterion for non-learners was negative or no discrimination between CS+ and CS- during the second half of acquisition (Lonsdorf et al., 2019). Therefore, the average difference between response levels of CS+ and CS- across acquisition trials 5-8 was calculated for the first interval responses of SCR and US expectancy respectively, and learners kept in the dataset were defined as participants whose average differences were bigger than zero. This resulted in an SCR learner dataset with 23 participants in groups standard and reversed extinction and 28 in the gradual extinction group and a US expectancy learner dataset with 31 participants in standard and reversed extinction and 33 in the gradual extinction group. The same analyses for extinction, extinction to renewal, and renewal were repeated in the new datasets, however, the results largely remained the same.

First Interval Electrodermal Responses

**Extinction**. Data indicated main effects of CS, *F*(1, 71) = 25.29, *p* < .001, η_p_^2^ = .26, and Block, *F*(11, 61) = 3.43, *p* < .001, η_p_^2^ = .38, where the response levels overall decreased across blocks (adjusted *α* = .004) and a Group × CS interaction, *F*(2, 71) = 10.99, *p* < .001, η_p_^2^ = .24. The response levels to CS+, averaging across blocks, were higher than those to CS- in groups gradual, *F*(1, 71) = 32.67, *p* < .001, η_p_^2^ = .32, and reversed extinction, *F*(1, 71) = 17.05, *p* < .001, η_p_^2^ = .19, but not in the standard group, *F*(1, 71) = 0.75, *p* = .391, η_p_^2^ = .01.

**Extinction to Renewal**. Analysis yielded main effects of CS, *F*(1, 71) = 5.18, *p* = .026, η_p_^2^ = .07, and Phase, *F*(1, 71) = 6.50, *p* = .013, η_p_^2^ = .08, such that response levels increased from the last extraction to the first renewal trials, and a Group × CS interaction, *F*(2, 71) = 4.81, *p* = .011, η_p_^2^ = .12. Averaging across phases, CS+ elicited higher levels of responses than CS- in the gradual extinction group, *F*(1, 71) = 14.36, *p* < .001, η_p_^2^ = .17, but not in the standard, *F*(1, 71) = 0.52, *p* = .472, η_p_^2^ = .01, or reversed groups, *F*(1, 71) = 1.24, *p* = .270, η_p_^2^ = .02.

**Renewal**. There were main effects of CS, *F*(1, 71) = 4.41, *p* = .039, η_p_^2^ = .06, and Block, *F*(1, 71) = 29.88, *p* < .001, η_p_^2^ = .30. The response levels were higher for CS+ than CS- and higher in Block 1 than Block 2. Effects involving the factor group were absent, all *F*s ≤ 2.26, *p*s ≥ .111, η_p_^2^ ≤ .06.

US Expectancy

**Extinction**. Data indicated main effects of CS, *F*(1, 92) = 323.87, *p* < .001, η_p_^2^ = .78, Block, *F*(11, 82) = 25.09, *p* < .001, η_p_^2^ = .77, and Group, *F*(2, 92) = 19.52, *p* < .001, η_p_^2^ = .30, and Group × CS, *F*(2, 92) = 30.95, *p* < .001, η_p_^2^ = .40, Block × CS, *F*(11, 82) = 5.53, *p* < .001, η_p_^2^ = .43, Group × Block, *F*(22, 166) = 7.06, *p* < .001, η_p_^2^ = .48, and Group × Block × CS interactions, *F*(22, 166) = 5.51, *p* < .001, η_p_^2^ = .42.

Following up the three-way interaction, a 12 (Block) × 2 (CS: CS+, CS-) ANOVA was conducted for each of the three groups, with a Bonferroni correction (*α* = .017). In the standard extinction group, there were main effects of CS, *F*(1, 30) = 22.81, *p* < .001, η_p_^2^ = .43, and Block, *F*(11, 20) = 18.81, *p* < .001, η_p_^2^ = .91. CS+ received higher US expectancy ratings than CS- and US expectancy ratings generally declined across blocks (adjusted *α* = .001). In the gradual extinction group, there were main effects of CS, *F*(1, 32) = 164.55, *p* < .001, η_p_^2^ = .84, such that the US expectancy ratings were higher during CS+ than CS-, and Block, *F*(11, 22) = 6.09, *p* < .001, η_p_^2^ = .75, such that the US expectancy ratings fluctuated across blocks (adjusted *α* = .001). In the reversed extinction group, there were main effects of CS, *F*(1, 30) = 164.77, *p* < .001, η_p_^2^ = .85, and Block, *F*(11, 20) = 10.11, *p* < .001, η_p_^2^ = .85, and a Block × CS interaction, *F*(11, 20) = 9.98, *p* < .001, η_p_^2^ = .85. CS+ received higher US expectancy ratings than CS- in all blocks, all *p*s < .001 (adjusted *α* = .001), with a mean difference of 42.32 in Block 1, dropping to 18.03 in Block 3, climbing up to 50.81 in Block 10, and decreasing to 32.23 in the last block.

**Extinction to Renewal**. Analyses indicated main effects of CS, *F*(1, 92) = 164.84, *p* < .001, η_p_^2^ = .64, Phase, *F*(1, 92) = 102.56, *p* < .001, η_p_^2^ = .53, and Group, *F*(2, 92) = 4.81, *p* = .010, η_p_^2^ = .10, and Group × CS, *F*(2, 92) = 13.18, *p* < .001, η_p_^2^ = .22, and a Group × Phase interactions, *F*(2, 92) = 9.94, *p* < .001, η_p_^2^ = .18. The focal three-way interaction was not significant, *F*(2, 92) = 2.40, *p* = .096, η_p_^2^ = .05. The Group × CS interaction was followed up with Bonferroni correction (*α* = .017) averaging over phases. Significant differences were found between CS+ and CS- in standard extinction, *F*(1, 92) = 10.22, *p* = .002, η_p_^2^ = .10, in gradual extinction, *F*(1, 92) = 101.69, *p* < .001, η_p_^2^ = .53, and in reversed extinction, *F*(1, 92) = 81.75, *p* < .001, η_p_^2^ = .47. The differential responses between CS+ and CS- were larger in the gradual (*M_diff_* = 31.32, *SD_diff_* = 21.55) and reversed (*M_diff_* = 28.97, *SD_diff_* = 15.77) extinction groups compared to the standard extinction group (*M_diff_* = 10.24, *SD_diff_* = 15.24), both *p*s < .001. The Group × Phase interaction was followed up with Bonferroni correction (*α* = .017) averaging across CSs. Significantly higher US expectancy was observed from the last extinction trial to the first renewal trial in standard extinction, *F*(1, 92) = 86.93, *p* < .001, η_p_^2^ = .49, gradual extinction, *F*(1, 92) = 12.33, *p* < .001, η_p_^2^ = .12, and reversed extinction groups, *F*(1, 92) = 21.49, *p* < .001, η_p_^2^ = .19, with a larger increase in group standard extinction (*M_diff_* = 27.44, *SD_diff_* = 18.63) than in groups gradual extinction (*M_diff_* = 10.02, *SD_diff_* = 16.60), *p* < .001, and reversed extinction (*M_diff_* = 13.64, *SD_diff_* = 13.50), *p* = .004.

**Renewal**. There were main effects of CS, *F*(1, 92) = 152.38, *p* < .001, η_p_^2^ = .62, and Block, *F*(1, 92) = 67.85, *p* < .001, η_p_^2^ = .42, and Group × CS, *F*(2, 92) = 4.47, *p* = .014, η_p_^2^ = .09, and Block × CS interactions, *F*(1, 92) = 10.26, *p* = .002, η_p_^2^ = .10. The Group × CS interaction was followed up with Bonferroni corrections (*α* = .017) averaging over blocks. There was significantly higher US expectancy during CS+ than CS- in standard extinction, *F*(1, 92) = 21.41, *p* < .001, η_p_^2^ = .19, gradual extinction, *F*(1, 92) = 73.26, *p* < .001, η_p_^2^ = .44, and reversed extinction groups, *F*(1, 92) = 67.93, *p* < .001, η_p_^2^ = .43, with the CS difference scores of standard (*M_diff_* = 14.93, *SD_diff_* = 18.73), gradual (*M_diff_* = 26.77, *SD_diff_* = 18.29) and reversed groups (*M_diff_* = 26.59, *SD_diff_* = 16.79) failing to reach significance after Bonferroni correction, all *p*s ≥ .029. The Block × CS interaction was followed up with Bonferroni correction (*α* = .025) averaging across groups. There were significant differences in US expectancy between CS+ and CS- in Block 1, *F*(1, 92) = 138.91, *p* < .001, η_p_^2^ = .60, and in Block 2, *F*(1, 92) = 129.81, *p* < .001, η_p_^2^ = .59, with a larger difference in Block 1 (*M_diff_* = 25.05, *SD_diff_* = 21.12) than Block 2 (*M_diff_* = 20.64, *SD_diff_* = 18.30), *p* < .001.
